# A Case of Bacterial Thyroid Abscess: A Rare Complication With Potentially Serious Consequences

**DOI:** 10.7759/cureus.39554

**Published:** 2023-05-27

**Authors:** Jafar Safarini, Omar R Khalil, Khalid J Faris, Mohammed A Aramin, Abdallah Al-Thaher

**Affiliations:** 1 Department of Surgery, Jenin Government Hospital, Jenin, PSE; 2 Internal Medicine, Al-Quds University, Jerusalem, PSE; 3 Department of Pediatric Surgery, Jenin Government Hospital, Jenin, PSE

**Keywords:** incision and drainage, neck swelling, bacterial, abscess, thyroid gland

## Abstract

Thyroid abscess is a rare but potentially serious condition that can affect young females. It is characterized by a localized collection of pus within the thyroid gland, often resulting from a bacterial infection. The occurrence of thyroid abscesses is a rare complication even in immune-compromised individuals. Nevertheless, when they do occur, they can present with symptoms such as neck swelling, pain, fever, and other systemic manifestations.

The diagnostic tool of choice for thyroid abscess is ultrasound, and the mainstay of treatment involves a combination of abscess drainage and antibiotics. In this case report, we describe the case of an 11-year-old girl who presented with neck swelling and pain and was subsequently diagnosed with thyroid abscess. The patient was successfully managed with incision and drainage, followed by a course of antibiotics.

## Introduction

Thyroid abscesses are a rare type of thyroid disease, accounting for only 0.1% to 0.7% of thyroid pathology. This is likely due to the thyroid gland's protective capsule, rich blood supply, and lymphatic drainage. Staphylococcus and Streptococcus are the most commonly reported bacteria causing this condition [1]. Several predisposing factors have been linked to its development, such as thyroglossal cysts, foreign objects in the thyroid due to esophageal perforation, immunosuppression, prior fine needle aspiration, and infection via blood or lymph [1].

Symptoms of thyroid abscesses typically include fever, neck pain, and a painful lump. Diagnosis is typically confirmed through a combination of clinical findings and diagnostic imaging, with ultrasound being the preferred initial investigation. The mainstays of management for thyroid abscesses include antibiotics and abscess drainage [2].

## Case presentation

An 11-year-old girl presented to our emergency room with acute onset of pain and swelling on the right and middle sides of the anterior and lower neck. The swelling started on the right side of the anterior lower neck and increased significantly in the next two days towards the midline. The pain was constantly dull, radiating to the right ear, exacerbated by lying supine, swallowing, and breathing. She also reported chills, fever, myalgia, generalized weakness, sleepiness, headache, dysphagia, odynophagia, loss of appetite, dysphonia, dyspnea, orthopnea, and choking sensation. She denied any history of night sweats, palpitations, paresthesia, chest pain, or change in weight, as well as any changes in vision, hair loss, constipation, or diarrhea. She had not yet experienced menarche and denied previous complaints of similar symptoms or any recent dental complaints or procedures. The history of the current illness began seven days prior to admission when the patient complained of a sore throat and discomfort with no history of fever. She sought medical help at a local health center and was prescribed antibiotics with no relief of symptoms. She then started to complain of right ear pain and increased severity of her symptoms, prompting her admission to the emergency room. Past medical history was insignificant, with no previous surgeries or exposures, and no known drug or food allergies.

On physical examination, the patient appeared ill and in moderate distress. Vital signs showed that the patient was febrile (39°C), with a blood pressure of 104/64 mmHg, a heart rate of 131 bpm, and oxygen saturation of 98% on room air. On inspection, there was asymmetric swelling of the lower and mid-neck on the right side, extending to the midline around 8 cm x 5 cm with no visible erythema or skin changes. The other side appeared normal with no obvious scars or discharge. On palpation, there was warm, highly tender swelling. The pain was severe, prohibiting further examination. No palpable preauricular, occipital, cervical, submental, submandibular, or supraclavicular lymph nodes were found. Chest examination showed no obvious signs of respiratory distress or obvious swelling, with no tenderness on palpation. Auscultation yielded normal vesicular breathing with tachycardia and no murmurs. Abdominal and neurological exams were both normal.

**Figure 1:**
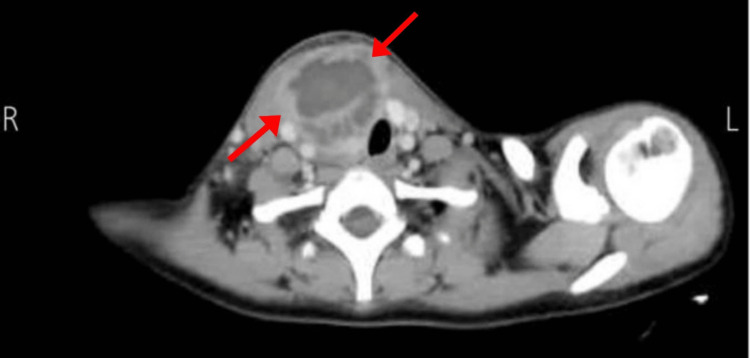
Neck CT scan showing the thyroid abscess (red arrows)

Initial laboratory results showed leukocytosis (17.3 WBCs/µL with neutrophils 85.8%) and elevated C-reactive protein (CRP) (216.5 g/L). Thyroid function tests revealed a low thyroid-stimulating hormone (TSH) of 0.0067 mIU/mL and a high thyroxine (T4) of 1.59 ng/dL. Urinalysis was clear with no signs of bacteriuria. Creatinine was 0.41 mg/dL, random blood sugar was 95.5 mg/dL, and electrolytes were recorded as follows: sodium (Na) 131 m/mL, potassium (K) 4 mmol/L. Ultrasound before admission showed a 3.7 x 2.2 cm right thyroid lobe abscess with an enlarged thyroid lobe and increased vascularity, as well as multiple enlarged cervical lymph nodes bilaterally. A neck CT scan was performed which showed a 7.3 x 4.5 cm multiseptated abscess in the position of the right thyroid, extending downward to the right and causing a compressing mass effect on the right jugular vein, as shown in Figure 1.

**Figure 2:**
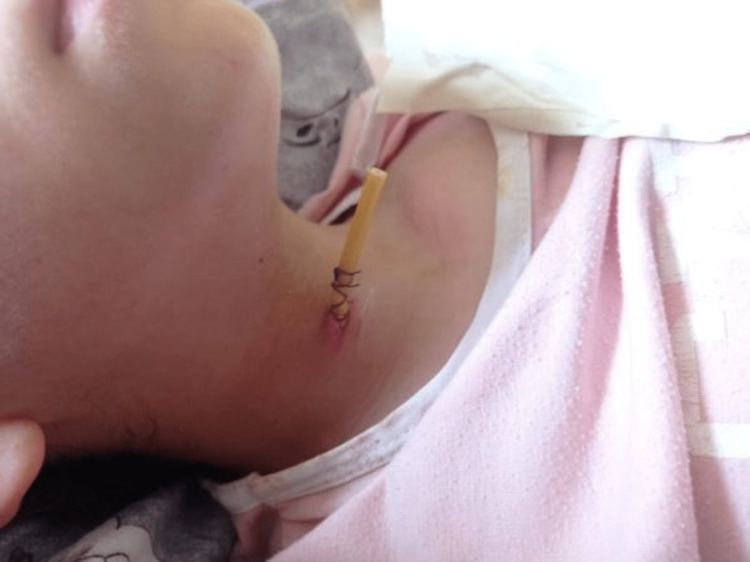
Penrose drain inserted through a well healed incision on postoperative day 2

After the diagnosis was confirmed, she was started on meropenem IV 300mg q8hr, and an urgent surgery was performed. A small transverse incision was made about 2 cm superior to the suprasternal notch, extending 1 cm in length. Open and dissection were performed from the midline to the right side, and a large amount of pus, around 400cc purulent discharge, was obtained. Cultures were taken, and washing with povidone-iodine solution and saline was performed. A drain was inserted as shown in Figure 2.

After the surgery, the patient was transferred to the ward and continued the treatment with meropenem and analgesics. The culture results were negative for any bacterial growth. Upon discharge (postoperative day 4), laboratory tests indicated no signs of inflammation, and the surgical wound appeared clean with no signs of erythema or discharge. An ultrasound was conducted which showed no fluid collection or abnormalities, allowing the removal of the drain. The wound was dressed and the patient was discharged on cefdinir and in good condition. On the follow-up visit one week after, she was in good condition with good wound closure; no masses or erythema were noted and she returned to her usual activity. 

## Discussion

Thyroiditis is classified into three primary types, namely subacute thyroiditis, Hashimoto thyroiditis, and acute suppurative thyroiditis [3]. Subacute thyroiditis is an immunological response of the thyroid gland that often occurs after an upper respiratory infection, believed to be caused by a viral infection. It is characterized by discomfort and pain in one or both thyroid lobes and is common among middle-aged women who have recently had symptoms of an upper respiratory tract infection after contracting ear, sinus, or throat viral infections like flu, mumps, or common cold [3]. Hashimoto thyroiditis, on the other hand, is caused by an immune response against the thyroid gland, resulting in chronic thyroiditis that typically leads to hypothyroidism over several months to years [4]. The most common pathway for thyroid infection is through the transmission of infectious agents via a pyriform sinus fistula. In the first decade of life, with a mean age of 7.6 years old, 80% of individuals with recurrent acute suppurative thyroiditis had a persistent pyriform sinus-thyroid fistula [5]. The cause of infection can be almost any microorganism, although Staphylococcus aureus, Streptococcus pyogenes, Staphylococcus epidermidis, and Streptococcus pneumonia are the most prevalent infectious etiologies. However, some rare cases have been reported where cultures have revealed Salmonella and Escherichia coli [5].

In order to confirm the presence of acute suppurative thyroiditis in the context of a pyriform sinus fistula, a fine-needle aspiration biopsy and tissue culture are necessary. Barium esophagography should be conducted during periods of quiescence to confirm the existence of a pyriform sinus fistula. The only successful therapy is the complete removal of the sinus tract [6]. Out of 1,309 successive thyroid surgeries, 117 patients were diagnosed with various forms of thyroiditis. Acute suppurative and non-suppurative thyroiditis are commonly observed after an upper respiratory infection and are characterized by discomfort and pain in one or both thyroid lobes. Patients also experience dysphagia, localized signs of inflammation, and occasionally an increase in temperature and heart rate. In six patients with acute thyroiditis, suppuration, and abscess development occurred. Once the patient responded sufficiently to medical interventions, such as the administration of antibiotics, chemotherapy, and supportive measures, drainage of the abscess was necessary [7].

A recommended imaging study for confirming the diagnosis of suppurative thyroiditis and/or an abscess is ultrasound, which is a first-line investigation. Ultrasound is a non-invasive imaging technique that does not use ionizing radiation and provides a clear view of the thyroid gland. Suppurative thyroiditis often shows a heterogeneous echotexture of the thyroid gland with a superimposed anechoic or hypoechoic mass on ultrasound as the abscess is forming. The echotexture of an abscess may change depending on the amount of internal debris or blood. Additionally, peripheral hypervascularity but little interval vascular flow is often observed in abscesses [8]. When there is no preexisting thyroid disorder, the thyroid function is typically within the normal range; however, there may be instances where hyperthyroidism or hypothyroidism is observed [3,4,8]. In certain cases, the destruction of thyroid follicles can lead to the release of thyroxine and triiodothyronine, resulting in thyrotoxicosis without the presence of hyperthyroidism, which is commonly referred to as transient hyperthyroidism [4]. This is the most plausible explanation for the thyroid function test results resembling hyperthyroidism observed in our patient. In the past, most patients with suppurative thyroiditis required open surgery, such as excision or incision and drainage, even with effective intravenous antibiotic treatment. However, a more conservative and less invasive approach has been found to reduce morbidity. In some documented cases, needle aspiration guided by ultrasound has also been effective in treating the condition [8].

## Conclusions

In conclusion, this report emphasizes the importance of considering bacterial thyroid abscess as a potential diagnosis in cases presenting with neck swelling or lump and signs of inflammation, even in the absence of predisposing factors, particularly in children. Thyroid ultrasound should be the first-line imaging modality, as it is highly efficient in detecting abscesses or collections and is non-invasive and easy to perform. Further investigations, such as CT scans or MRI, should be reserved for cases where ultrasound was not informative or when other etiologies are suspected. Early diagnosis and treatment with abscess drainage and antibiotics are crucial for a favorable outcome. Physicians should be aware of this rare but potentially serious complication of thyroiditis and include it in their differential diagnosis when evaluating patients with neck swelling and signs of inflammation.
